# Screening strategies and production of biosurfactants (BSs)/bioemulsifiers (BEs) from marine yeasts and fungi

**DOI:** 10.3934/microbiol.2025023

**Published:** 2025-07-18

**Authors:** Surekha K. Satpute, Ibrahim M. Banat, Samadhan R. Waghmode, Shrikant Hulkane, Mahima Bagayatkar, Riddhi Chakraborty

**Affiliations:** 1 Department of Microbiology, Savitribai Phule Pune University, Pune, Maharashtra, India; 2 School of Biomedical Sciences, University of Ulster, Coleraine, BT52 1SA, N. Ireland, UK; 3 Department of Microbiology, Elphinstone College, Fort, Mumbai, India

**Keywords:** Antimicrobials, biosurfactants, bioemulsifiers, fungi, GRAS, screening assays, Yeast, renewable substrates

## Abstract

The unique characteristics of the marine ecosystem support the existence of microorganisms with exceptional metabolic potential, enabling them to produce high-value bioactives. Among these, biosurfactants (BSs) and bioemulsifiers (BEs) are notable multifaceted molecules, distinguished by their unique structural, molecular, and functional properties. Marine yeasts and fungi produce BSs/BEs with distinctive properties in terms of stability under extreme conditions. It is important to mention here that in comparison to marine bacteria, yeast and fungi of the same habitat have been explored only intermittently. Some of the BSs/BEs producing bacteria may prove to have some pathogenic or cytotoxic traits or components, while most yeasts are mainly classified as Generally Recognized As Safe (GRAS) (by the Food and Drug Administration-FDA, USA), making their BSs/BEs products more amenable for a wide range of applications. The diverse and unique potential of surface-active agents is further enhanced by the endosymbiotic associations often found in marine yeasts and fungi. These microorganisms are acknowledged to produce glycolipidic (rhamnolipids, sophorolipids, and mannosylerythritol lipids) or glycolipoproteins. The SL have been reported well for their strong antimicrobial activity, including effectiveness against drug-resistant pathogens, making them promising candidates for controlling foodborne pathogens in the food industry. Furthermore, these microorganisms can utilize a broad range of carbon sources from simple substrates, like glucose and glycerol, to complex feedstocks such as food, oil, agricultural waste, and wastewater, which not only support their growth but also promote the production of substantial yields of these BSs/BEs. In this review, we endeavor to explore BSs/BEs from marine yeasts and fungi, including the screening, characterization, identification, production, and importance.

## 1. Introduction

Marine environments possess their own uniqueness with respect to the presence of high salt content, particularly NaCl ≈ 3.5–4.0% along with several other salts. Hypersaline systems, deep–sea oceans, hydrothermal vents, and coastal water represent some of the popular examples of marine aquatic bodies. Both biotic and abiotic factors impact the survival of life in the marine environment. Consequently, the marine microorganisms produce several bioactive compounds, including biosurfactants (BSs)/bioemulsifiers (BEs). Thus, the marine biosphere becomes a wonderful resource to acquire highly commercially valuable byproducts. The amphiphilic nature with varied anionic, cationic, and non–ionic groups encourages their applicability for diverse applications. The hydrophilic and hydrophobic moieties enable the dispersion of insoluble phages together and provide freedom to explore many more applications in the industrial sector. Surfactant molecules reduce the surface tension (ST) and interfacial tension (IFT), whereas emulsifiers permit miscibility of different liquids (oil and water), which otherwise are insoluble in nature. Emulsifiers may not reduce the ST commendably; however, they are crucial in the emulsification process [1]. Petroleum-based products are employed in the manufacturing of chemical surfactants; however, their toxicity and non-biodegradable nature are major concerns [2]. Thus, in recent times, curiosity toward BSs/BEs has markedly increased [3]. Additionally, several powerful functional properties, viz, emulsification, ST, IFT, wetting or spreading, reduction in viscosity, detergency, cleansing, and foaming, are truly appreciated.

The BSs/BEs producing marine microbial communities have been reported from both unpolluted and polluted oceanic sites [4]–[10]. It is important to mention here that the common interest of the research community appears towards reporting overall applications of BSs/BEs [1],[3],[11],[12]. The sparse literature provides the marine microbial origin of BSs/BEs [13]–[19]. Again, due to the negligible work documented on yeast and fungal origin BSs/BEs, not many dedicated review articles are found in the literature. The distinctiveness of this review lies in its inclusion of the comprehensive information published over the past 15 years, focusing on the discovery of marine yeast and fungi or the production of BSs and BEs, along with their potential applications. Strategies that can be employed to select a particular screening method have been recommended. Thus, we aim to guide researchers in choosing precise screening techniques for identifying BS/BE-producing marine microorganisms. This article also sheds light on diverse yeast and fungal strains that have been documented for the production of surface-active agents, along with fermentation details. Information regarding structural characterizations and stability under varied conditions has been included. The role of cheap, renewable substrates and statistical tools employed in enhancing the yield of BSs/BEs from marine yeast and fungi has also been emphasized.

## 2. Literature search strategy

A comprehensive and methodical literature survey was carried out using several online platforms/databases such as Google Scholar, PubMed, The National Center for Biotechnology Information (NCBI), ScienceDirect, Web of Science, Directory of Open Access Journals (DOAJ), and Journal Storage (JSTOR) to categorize appropriate review and research articles published to date. Appropriate keywords were employed during the literature search. Peer-reviewed articles, journals, patents, and books published by reputable sources such as AIMS Press, Elsevier, Springer Nature, Frontiers, Wiley, Multidisciplinary Digital Publishing Institute (MDPI), Oxford University Press, Taylor & Francis, SAGE Publications, American Chemical Society (ACS), The Royal Society of Chemistry (RSC), Bentham Science, BioMed Central, and Hindawi and others were consulted.

## 3. Unveiling the uniqueness of marine ecosystems, acquaintance with microbial life, and screening assays employed in identifying potent biosurfactant/bioemulsifier producers

Around 71% of the Earth's surface is covered by water, of which seawater accounts for 97.5% [20]. Of all the dissolved chemical species in seawater, Na^+^ and Cl^-^ account for 86% [21]. Other parameters, including depth, latitude, bottom topography, oceanic currents, wind, aerosol deposition, weathering of rocks, riverine inputs, and submarine groundwater discharge activities, contribute to the physicochemical properties of the oceans. However, the marine waters have been undergoing dramatic changes in recent decades due to ongoing climate change [22],[23]. For instance, temperatures increase water column stratification, preventing subsurface nutrients from reaching surface waters and affecting marine ecosystems. Higher water temperatures also affect the oxygen solubility, thereby reducing the dissolved oxygen content and leading to the expansion of oxygen minimum zones [22],[23]. Another major challenge is the high concentration of dissolved CO_2_ in the atmosphere, which is consequently absorbed by oceanic waters, leading to a reduction in pH, a process termed ocean acidification [24]. This process has a direct effect on marine life (microorganisms, plants, and animals). How these processes affect the marine bioresource potential is not fully understood [25],[26].

The existence and survival of microbial communities, animals, and plants within the vast marine environment are linked to the physical and chemical composition of their surroundings [27]. The marine biosphere harbors affluent microbial communities with immense biodiversity that is worth exploring for the benefit of mankind. So far, our perception of spatial patterns of microbial and functional diversity, and their exploitation for natural products, is quite limited [28]. Isolating particular microbes from the marine biosphere can be challenging compared to the terrestrial environment [29]. Consequently, the inclusion of High-throughput screening (HTS) for culturing marine microbes is needed to deal with the ‘Great Plate Count Anomaly (GPCA)’ challenges [30],[31]. Staley and Konopka [32] presented the concept of ‘GPCA’, which represents differences between the count of microbial load and the number of microorganisms in each environment, and the actual microbial load or the cells that could be cultured under laboratory conditions. Complex relationships between diverse microorganisms and the surrounding environment are difficult to simulate in laboratories.

The chances of culture contamination during marine microbial fermentations can be quite low due to unique cultivation conditions (halophilic, thermophilic, and acidophilic or alkalophilic) [33]. Therefore, some modifications might be required to grow certain isolates in unusual habitats. For example, supplementation of a special growth medium (Zobell Marine Medium), higher NaCl concentration (up to or >3.5%), and certain conditions (pH, temperature, or pressure) are needed to mimic the marine environment [34]. Diverse microbial communities of marine origin are involved in the production of distinct classes of BSs/BEs. Scientists working on BSs/BEs use numerous screening assays to identify potential candidates. Most of the methods involve detecting the presence of surface–active agents in the cell-free supernatant (CFS). Some of the screening techniques use characteristics like the hydrophobic nature of the cell surface to identify potential BS/BE producers. Recently, the focus seems to be on developing automations in screening technologies to reduce the burden of manual work. These efforts have resulted in developing HTS approaches. Imaging and spectroscopic techniques can be used for BSs/BEs. However, most of the time, pure BS samples can be explored for demonstrating the applications as an anti–adhesive and biofilm inhibiting or disrupting agent [35]. Different screening methods are truly supportive in identifying the potential BS/BE producers [36].

**Figure 1.**
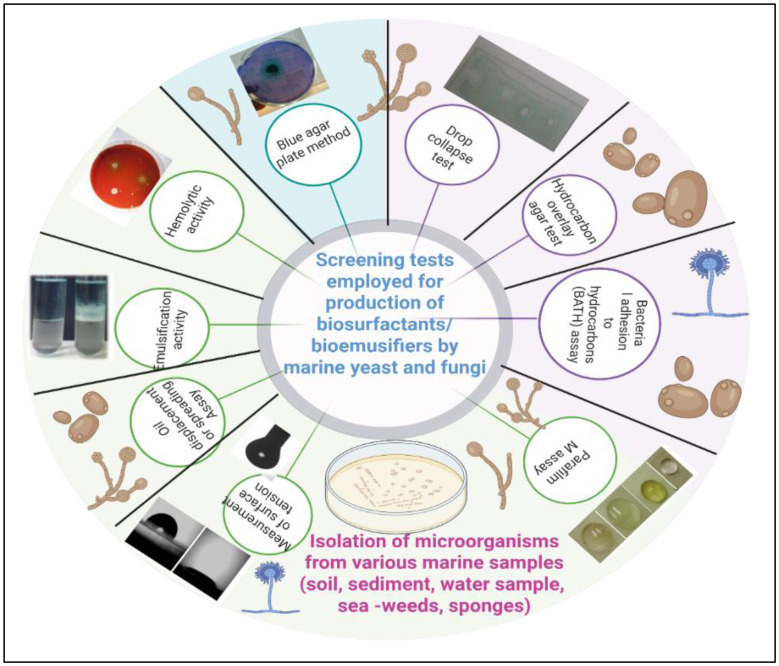
Different screening techniques employed in identifying biosurfactant/bioemulsifier producers.

The properties of BSs/BEs are being explored through several screening assays, like 1. Hemolytic assay (HA), 2. Drop collapse test (DCT), 3. Oil displacement assay/test or Oil spreading assay/test (ODA/ODT or OSA/OST), Bacterial adhesion to hydrocarbons (BATH) assay, 5. Hydrocarbon overlay agar test (HOAT), 6. Blue agar plate assay (BAPA), 7. Emulsification tests or emulsification index (EI), 8. Parafilm M assay, and 9. ST measurements [37],[38], which are represented in Figure 1. Each of the screening assays has its own limitations (false positive or negative results). The details of the screening techniques followed by investigators are generally as follows:

## 4. Screening assays employed in detecting potent biosurfactant/bioemulsifier producers

### 4.1. Hemolytic assay (HA)

The qualitative assay necessitates inoculation of culture(s) on a nutrient agar (NA)/Luria Bertani (LB) agar medium supplemented with sheep blood 5% (v/v) and NaCl (≈3.5%). Minimal Salt Medium (MSM) supplemented with NaCl or Zobell Marine Agar (ZMA) with blood is used to maintain the salt requirement of marine cultures. The presence of high amounts of salt encourages the growth of halophiles and possibly hinders the growth of non–salt–tolerant strains. Inoculated cultures are incubated further and examined for hemolytic activity [39]. Results are interpreted in three types of hemolytic reactions around the culture; 1. Alpha hemolysis–Green or brown zone; 2. Beta hemolysis–Transparent/clear zone; and 3. Gamma hemolysis–No noticeable hemolysis. This technique assesses whether an organism can produce appreciable quantities of BSs when grown on carbon sources other than hydrocarbons [40].

### 4.2. Drop collapse test (DCT)

This is one of the rapid tests due to its simplicity and minimal requirements. The presence or absence of surfactant is indicated by the collapse or beading of the droplet, respectively. In the quantitative version, the diameter of the droplet after a specific time period can be measured to detect the surfactant. The microbial culture is inoculated in MSM supplemented with appropriate nutrients. The drop of the culture broth can be visualized better by adding methylene blue (5 µL) without altering the original shape of the droplets [41]. This captures images that differentiate the control and the test samples. High salt concentrations may linearly affect the ST of the CFS [42]. The DCT is usually reproducible, which is important for obtaining consistent results across experiments [43]. Due to limited sensitivity, it is not suitable to accurately detect low levels of BSs in the culture broth, and it is not suitable or useful for the identification of molecules involved. Interference from other compounds may also limit the applicability of this technique [43],[44]. The use of a microtiter plate enables the simultaneous screening of multiple samples, making it amenable to HTS approaches.

### 4.3. Oil displacement assay/test or oil spreading assay/test (ODA/ODT or OSA/ODT)

This test was introduced by Morikawa et al. [45] and became popular due to its ease. Researchers can overlay approximately 10 µL of hydrocarbon or crude oil over the surface of distilled water (40 mL) taken in a petri dish. Subsequently, approximately 10 µL of test culture broth or its CFS is gently added over the oil layer formed on the water surface, and the area of the clear zone is measured. Like other techniques, the addition of salt to water can enhance the wetting property of the solution. This means that the saltwater might spread more easily over the surfaces as compared to the control (non–salty water). This effect is due to a decrease in ST of saltwater or seawater compared to pure water [46]. The presence of ions in saltwater can also lead to ion–dipole interactions with molecules in the oil and may affect the behavior of oil droplets in saltwater and their spread. Depending on the type and concentration of salt, electrostatic effects can transpire and affect the system. These effects might alter the forces between molecules at interfaces, including those between water, oil, and air [46]. The ODA or OSA has some advantages by measuring the oil–spreading ring's diameter, as it enables precise quantitative analysis of BSs, ensuring a dependable and reproducible approach. The simplicity and speed of ODA or OSA make it accessible for routine screening, with recent refinements for enhancing accuracy and stability [47] in addition to being an eco–friendly approach [48].

The ODA/ODT has some challenges, like constrained applicability, as it may not effectively identify BSs with diverse mechanisms of action or those optimized for different environments. Additionally, the sensitivity of the method to environmental conditions, including temperature, humidity, and oil types, can compromise the accuracy and reproducibility of results [47],[49]. The semi–quantitative nature of the technique, based on the diameter of the spreading ring, does not estimate the concentration of BS and lacks linearity. Furthermore, the method lacks specificity, providing limited insight into the structural details of the BS molecule. Reliance on a single endpoint measurement may oversimplify the complex nature of BS's activity. The potential for false-positive or negative results due to factors like microbial growth and sample interactions could introduce inaccuracies. Scaling the technique for HTS might necessitate exploration of significant resources [49]. Last, the subjectivity involved in visually interpreting oil spreading patterns introduces an element of variability in the results. Despite these drawbacks, ODA/OSA remain a valuable screening tool when used in association with other complementary techniques [38]. The screening assays can be updated for automation and enhance the potential to identify novel strains with distinct BS production properties [44].

### 4.4. Bacterial adhesion to hydrocarbons (BATH)

This is another simple and indirect test for assessing BSs/BEs producers. Microbes possess a unique ability to adhere to various hydrocarbons, which also indicates the hydrophilic nature of bacteria resulting in the formation of an emulsion. Consequently, it demonstrates the formation of an emulsion in the presence of a surface-active agent [50]. The cell pellets of a test organism are washed and suspended in a buffer solution, which is diluted further using the same buffer to achieve ≈0.5 optical density (OD) at 610 nm. About 2 mL of cell suspension is added to crude oil (10 µL) and mixed thoroughly using a vortex (3 min). After mixing, phase separation (crude oil and liquid) is achieved by letting the tubes stand still for 1h. Followed by visible separation of phases, the OD of an aqueous phase is examined using a spectrophotometer. Further, the cells attached to crude oil are calculated as the percentage (%), denoting the adherence of bacterial cells to the hydrocarbons (H), using the following formula:

$$H=\left(1-\frac{A}{A_{0}}\right)\text{ X }100\%$$

Here, ‘A’ denotes the absorbance after mixing of the cell suspension with the hydrophobic phase; ‘A_0_’ denotes the absorbance of the bacterial suspension in the absence of the hydrophobic phase. While carrying out the BATH assay, researchers usually use a high salt concentration to grow the marine bacteria. High salt concentrations can promote the aggregation and precipitation of proteins (“salting–out” phenomenon). This occurs due to the disruption of hydration barriers between molecules, causing hydrophobic regions to be exposed and leading to attraction forces between neighboring molecules. BATH has been modified as Microbial Adhesion to Hydrocarbons ‘MATH’ [51]. In the BATH assay, the hydrophobic interactions between bacteria and hydrocarbon substrates are crucial for adhesion. Alterations in the salt concentration could affect the degree of hydrophobic interaction and thus influence bacterial adhesion [52]. Moreover, small changes in NaCl concentration may have a strong effect on the adhesion of bacteria to surfaces. While this observation is not directly related to hydrocarbon substrates, it highlights the sensitivity of bacterial adhesion due to the alteration in salt concentration. This assay, however, has several advantages over other methods, including its simplicity, low cost, and freedom to screen large numbers of samples in a relatively short time [53],[54]. The BATH assay can be affected by the contaminants present in the culture medium, which interferes with the results. It is, however, an important complementary method to confirm the results achieved by other assays [55],[56].

### 4.5. Hydrocarbon overlay agar technique (HOAT)

In this technique, ZMA plates are coated or overlaid individually with crude oil, kerosene, or hydrocarbons (hexadecane, benzene, toluene etc.). Freshly grown pure culture is spotted on those media plates and incubated up to one week at 28 °C. A colony exhibiting a halo around it is considered positive for the production of BSs. This visual detection simplifies interpretations in a better way. Furthermore, the method enables rapid screening of a large number of microbial isolates, making it efficient for initial assessments. Literature suggests that certain microorganisms may exhibit enhanced BS production and hydrocarbon degradation under specific salt concentrations. Sharma et al. [57] indicated that certain BSs–producing strains exhibit stable emulsification activity over a wide range of salt concentrations (2–7% NaCl). The HOAT is simple, cost–effective, and does not require sophisticated equipment, making it an accessible option. HOAT provides qualitative information about the presence of BSs, aiding in preliminary evaluations and strain selection without precise quantitative data for production. False positive results can arise due to a depletion of nutrients, which can mislead the results. Conversely, false negative results can occur when certain BSs fail to generate halos (slow diffusion or interactions) on the plate due to insufficient amounts. The technique does not offer insight into emulsification capacity or stability. Moreover, the visual interpretation is subjective, introducing potential variability in result interpretation. These limitations underline the need for careful consideration and complementary techniques while using the screening test [37],[58].

### 4.6. Blue agar plate assay (BAPA)

This is a semi–quantitative assay that facilitates the detection of extracellular glycolipids or other anionic BSs. MSM supplemented with different carbon sources (e.g., glucose, fructose, peptone, maltose, and galactose) and trace elements solution (TES) are used in this assay. Further, cationic surfactant–cetyltrimethylammonium bromide (CTAB) and the basic dye–methylene blue (MB) are added. Both CTAB and MB form ionic pairs that enable the detection of glycolipid BS producers in the form of dark blue halos around the culture indicate a positive test [59],[60]. Thus, these methodologies are useful for growing the specific cultures and identifying potential glycolipid BS producers. This method offers a cost-effective screening approach. Positive results can guide researchers to select appropriate carbon sources for large-scale BS production processes. It also permits relatively quick evaluation of glycolipid BS production, which can be advantageous in screening for many isolates. The method, however, lacks precision in quantifying BS amounts and may not accommodate various types of BS, limiting its range [61]. Quantitative data on yields and properties of BS beyond detection are limited and provide no insights into the emulsification capacity, stability, or specific applications of the BS. There is a potential for false-positive/negative results due to subjective visual interpretation of dark blue halos. Moreover, it might not be ideal for HTS, as manual observation and semi–quantitative nature could be time–consuming [53],[61].

### 4.7. Emulsification test or Emulsification index (EI)

This simple technique measures an emulsification activity due to the dispersion of one insoluble phase into another in the presence of BEs [62]. The surface–active agents enable interactions between insoluble phases and form stable emulsion (oil–water). The assay is usually performed by adding kerosene to the aqueous fraction of the test organism. The blend is vortexed at high speed for 2 to 3 min. The tubes are undisturbed, and EI is calculated from the height of the emulsified layer formed and its stability over time 24 h (E_24_) or 48 h (E_48_). EI is denoted as the ratio of the height of the emulsified layer and the total height of the liquid. Emulsification processes are significant in microbial enhanced oil recovery (MEOR) [63]. The substrates used for the growth of an organism and the production process may influence the emulsification abilities. Surface active agent permits bio-accessibility of hydrophobic or water-insoluble substrates that are essential for the survival of microorganisms under extreme conditions [63]. The overall microbial system, class of surface-active agents, along with environmental conditions, play a significant role in the emulsification of insoluble liquids. Microorganisms isolated from oil–contaminated environments are always advantageous as BS/BE producers and can be employed for bioremediation-related applications [64]. Emulsification has a key role in identifying favorable conditions that can be of benefit for the food and cosmetic industries and can contribute to selecting comparatively more stable emulsions [65]. The microscopic analysis of emulsified droplets formed by the presence of surfactants and immiscible liquids is useful in designing powerful formulations. However, emulsifications are also associated with certain challenges. Instability and phase separation may lead to inaccurate emulsion formation. This assay is quite labor–intensive, and interpretations can be subjective. Specialized equipment like high–end microscopy (to visualize micro–droplets) and expertise are needed for efficient analysis. Emulsification assays may not predict the real behavior of BSs/BEs. Moreover, variability in emulsification activities may hamper the reproducibility of the findings, and some emulsions might not be suitable for standard assays. BEs are not BSs and necessitate diverse screening approaches [66]. Researchers should consider these factors before drawing meaningful conclusions on the use of BSs/BEs for intended applications.

### 4.8. Parafilm M Test

In this test, BS destabilizes a drop of liquid (polar) through a reduction in ST and the IFT existing between the drop (hydrophilic) and the parafilm strip (hydrophobic surface). The flattened shape of the drop indicates a reduction in ST and IFT, whereas the original dome shape can be seen in the absence of surfactant in the test samples [67]. A minute after the culture broth or CFS is placed on a parafilm strip at room temperature, the drop (shape and diameter) is observed [68].

### 4.9. Metagenomic approach and High-throughput screening (HTS) approach

This is a commanding approach for the screening of BSs/Bes-producing microbes, particularly those that are not able to grow under laboratory conditions. The metagenomics approach offers immense opportunities to explore BSs/BEs from microbes that are challenging to cultivate from exotic and untapped environments. The process initiates with the collection of nucleic acids from the samples (sediments, soil, water, etc.) collected to detect BS-producers along with their respective genes. To unlock the unique biochemistry within the ‘Viable But Nonculturable Cells' (VBNC), support from the culture–independent approaches is imperative, and thus, the implementation of metagenomics approaches comes into play, serving as a crucial strategy for discovering novel compounds within these not-yet culturable bacteria [69]. Traditional microbial cultivation methods are bypassed to harness the diverse microbial communities efficiently. Taxonomic and functional genomic analysis are supported through databases and several computational approaches (NCBI, The Basic Local Alignment Search-BLAST), Clusters of Orthologous Groups of Proteins (COG), BSs and Biodegradation Database–BioSurfDB, Antibiotic and secondary metabolite analysis shell, Kyoto Encyclopedia of Genes and Genomes–KEGG, etc. [70]. Understanding microbial communities, their genes involved in BS/BE production, and their identification and characterization can facilitate the selection of potential microbes for intended applications. Comprehensive genome analysis of the microbes enables competent identification and screening of genes along with their taxa involved in the production of BSs/BEs [71].

Despite the availability of several screening protocols, the strategic HST–based screening would be a wiser approach to tap the unculturable strains. In sequence–based metagenomics, the genes or gene clusters are examined through the sequencing of polymerase chain reaction amplification, identifying regions with similarities to known genes or biosynthetic pathways involved. Functional metagenomics involves cloning randomly sheared environmental DNA into a suitable vector, utilizing native promoters to detect clones possessing desired activities. Screening assays developed for identifying BS producers can be adapted for functional HTS with large environmental clone libraries [72]. The BS protein has been documented from a metagenomic library. In 2020, Araújo et al. [73] constructed a metagenomic library and showed the presence of new BS-producing as well as hydrocarbon-degrading genes in the extracted DNA from the soil samples collected from Jundiaí River, Brazil. Initial functional screening led to the identification of a clone responsible for the production of BS protein and showed the presence of an open reading frame (ORF) having sequence similarity with the hypothetical protein belonging to family Halobacteriaceae. They also purified the protein (named as MBSP1) and demonstrated its BS activity. Additionally, the strain *E. coli* RosettatM (DE3) was transformed with the MBSP1 clone which improved its abilities for the degradation of aliphatic hydrocarbons. The study stated a remarkable finding: The presence of a single gene encoding a protein having substantial tensioactive properties that can be expressed in a host cell (*E. coli*) even in the absence of substrate [73].

**Figure 2.**
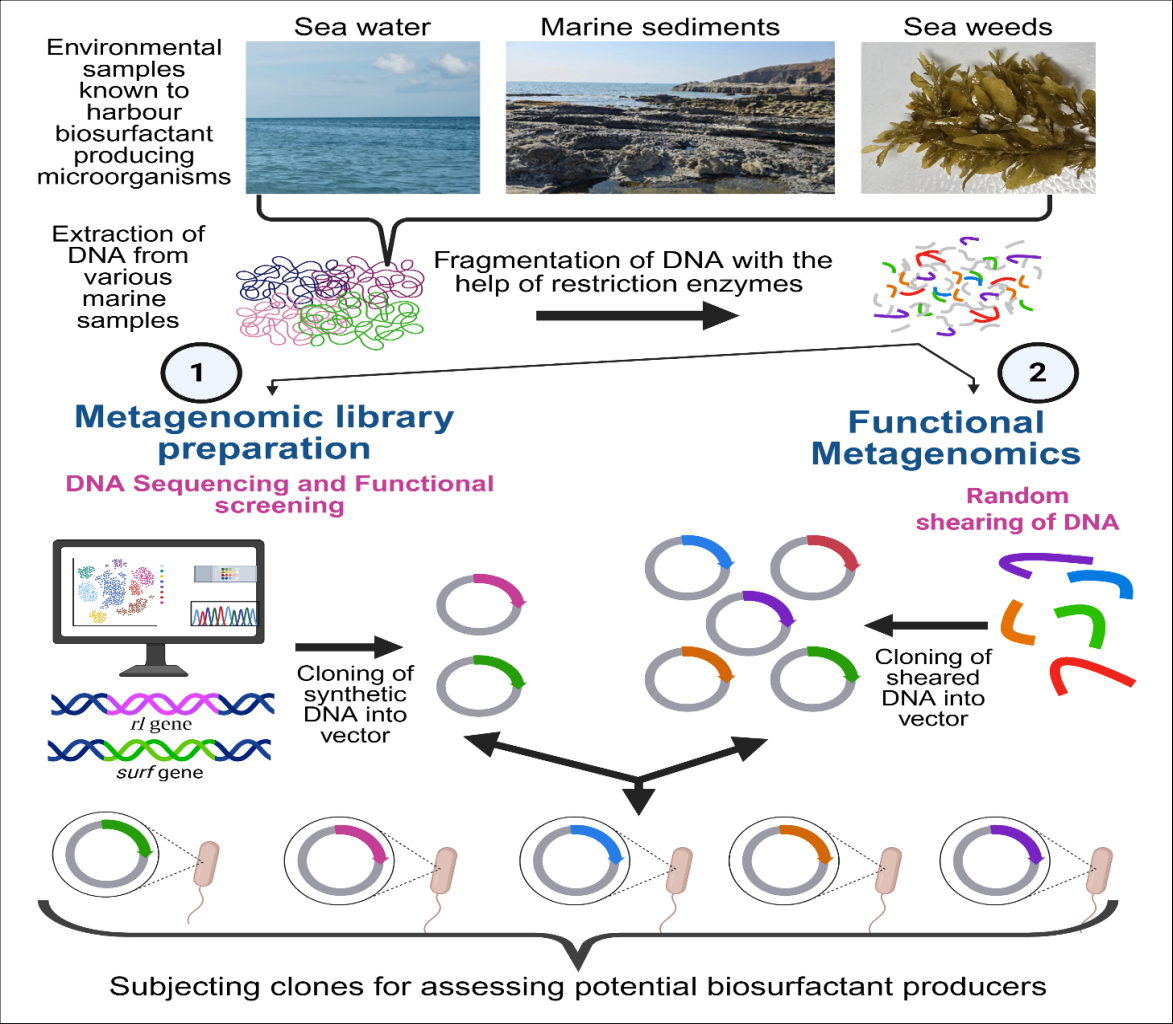
High‑throughput screening for biosurfactant production via the metagenomic approach.

In 2019, Williams et al. [74] explored uncultured bacteria and constructed a metagenomic library with DNA extracted from the environment directly in *E. coli* as a model system. Another multiple hosts, *Pseudomonas putida* and *Streptomyces lividans*, were also included as shuttle vectors. Further, the library was screened in both bacterial systems (*E. coli* and *P. putida*) through the paraffin spray assay. The group reported a clone of *P. putida* for BS activity. Analysis of sequences and transposon mutagenesis authenticated the role of ornithine acyl-ACP N-acyltransferase in surfactant activity. Furthermore, overexpression of the olsB gene was accomplished to produce lyso-ornithine lipid along with BS activity in the CFS under the control of the T7 promoter. Overall, Metagenomic DNA analysis explores a sequence and a functional activity screening approach. The HTS approach is progressing where automation and miniaturization assist the screening for detecting BSs/BEs producers from a huge number of samples, which can be explored further in the large-scale production of surface-active agents [44]. Figure 2 represents a pipeline overview for HTS for BS production via the metagenomic approach.

## 5. The structures and applications of marine yeast and fungi in the origin of biosurfactants/bioemulsifiers

**Figure 3.**
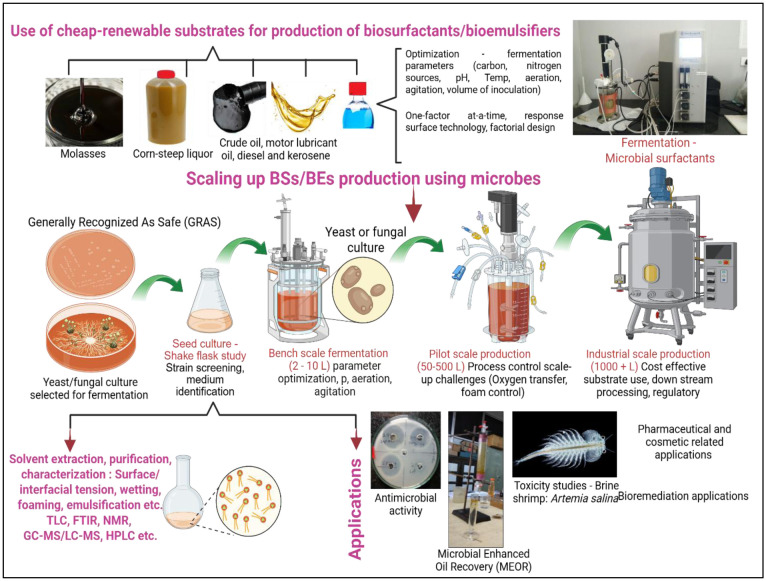
Overall protocol employed in the production and purification of biosurfactants/ bioemulsifiers from microorganisms, along with applications.

Habitats for isolation of marine microorganisms include the sea, hypersaline habitats, oceans, Antarctic areas, and salty brine, whereas non-marine sources include freshwater bodies, soil, industrial effluents, air, waste from sewage, oil wells, and refineries. It is very challenging to isolate particular microbes from marine water bodies since there is a dynamic and complex environmental condition. The probability of isolating a certain type of microbial population is high from sources other than marine water bodies. Simple media can support the growth of microbes from non-marine sources, compared to marine bodies. Maintaining a particular environment and the physiological conditions for the growth of marine microbes is quite demanding. The difficulty in maintaining humidity, salinity, and with other extreme conditions, makes it challenging to grow the marine microbes in the laboratory. However, there are certain advantages in using these marine microbes for BSs/BEs when compared to other or non-marine microbes. The ability of marine microbes to thrive under extreme conditions of pH, salinity, and temperature opens enormous opportunities to explore novel bioactive compounds and molecules for industrial applications. While working with marine microbial BSs/BEs fermentation processes, the chances of contamination are quite low since organisms are grown in the presence of high salt concentrations. Marine habitats harbors untapped microbial diversity and thus present an extensive genetic pool to discover and engineer novel BSs/BEs. The novelty in structures of yeast or fungus–originated BSs/BEs is drawing strong attention from the scientific community from industrial perspectives. Consequently, in recent times, there is a continuous increase in the number of research articles published describing the production, characterization, and applications of surface–active compounds by yeasts and fungi from diverse marine habitats. Usage of yeast in BSs/BEs production is quite interesting since it is a ‘Generally Recognized As Safe’ (GRAS) system [75], posing the freedom to harness them for broader applications [76]. Furthermore, protocols employed in the production, extraction, and purification of BSs/Bes, along with their applications, are represented in Figure 3.

Several yeast and fungal strains have been isolated from marine sampling sites contaminated with oil or uncontaminated sediments, soil, and water samples [77]. Sometimes, marine weeds and animals are habituated by yeast and fungi and have therefore been used as samples to isolate BSs/BEs producers. The literature has been discussed as follows: de Oliveira Barros et al. [78] isolated 19 yeast cultures from the zoanthids (soft corals), which were screened for the production of BS through emulsification tests. The screening assay showed a ≥50% EI by five isolates. Along with the emulsification test, some of the other methods included viz, ODT, parafilm M assay, DCT, and ST measurement. The tolerance of produced BSs for environmental conditions was also analyzed. Fermentation parameters like carbon source, temperature, pH, and salinity were also included. These results demonstrate that BSs extracted from yeast can emulsify hydrocarbons under different physicochemical conditions and metabolize kerosene as a carbon source. Among the 19 isolates, *Y. lipolytica* LMS 24B shows production of glycoprotein complex with low lipid content. Along with surfactants, microbial biomass also has other biotechnological potentials as emulsifying agents. Analytical characterization through Fourier Transform Infrared Spectroscopy (FTIR) shows the functional groups of glycoprotein-based BS. de Oliveira Barros and coworkers [78] isolated intracellular BS, where the cell pellet was used to measure the EI and the yield of biomass (g/L). They expressed the yield as an EI, where 900 mg cell wall/L, and the diluted one (5.78 mg/mL) result in 61.2% of EI, and demonstrates that the BS that adheres to the cell wall of yeast is suitable for emulsification activity. Thus, the yield of BS reported is related to EA. LMS 24B is one of the best yeast models to isolate BS, where EI (61.2%) is obtained even at a high temperature (120 °C). In view of the status of developing new bioproducts and bioprocesses, these findings bring a new perspective to BS production by marine yeasts, especially *Y. lipolytica*. In particular, the presented results validate the relevance of marine environments as valuable genetic resources, where yeast strains are capable of metabolizing and emulsifying petroleum derivatives. Research on *Y. lipolytica* IMUFRJ 50682 by Fontes et al. [79], optimized the production of BS through a factorial design approach. Haegler et al [80] isolated strain IMUFRJ 50682 from an estuary (Guanabara Bay, Rio de Janeiro, Brazil). Fontes et al. [79] optimized the BS production process by correlating through EI and ST measurements. The response surface method (RSM) is an efficient tool in improving the media components. This work showed high EI (81.3%) and effective reduction in ST (19.5 mN/m) by the produced BS.

*Candida bombicola* is one of the remarkable yeast strains that produces the SL class of BSs that has innumerable applications in the pharmaceutical, cosmetic, and food industries. *C. bombicola* also shows great potential in environmental bioremediation. Strain *C. bombicola* has been reclassified and is now known as *Starmerella bombicola*. Pinto et al. [81] worked on *C. bombicola* for the production of glycolipid BS by growing it in a fermentation medium supplemented with low-cost substrates like sugar-cane molasses, frying oil waste, and corn steep liquor (CSL). BS was produced at different levels with progressive yield at Flask, bioreactor (1.2 L), and bioreactor (3.0 L) of 12.5, 19.8, and 61.0 g/L, respectively, indicating significant improvement during the process. The scale-up studies were performed up to a 50 L bioreactor with a yield of 221.9 g/L. The toxicity study showed no harmful effects on *Artemia salina* and *Allium cepa* (onion). Moreover, *A. salina*, popularly known as brine shrimp, which is used as a model organism for preliminary toxicity studies. The ease of cultivation of the brine shrimp, its rapid hatching, and its sensitivity to toxic substances encourages its use as a model organism for scientific toxicity-related aspects. It is particularly valuable for screening a large number of extracts, like those from medicinal plants, where aseptic techniques are not required, making it a cost-effective and rapid alternative to more complex assays.

Nimsi and Manjusha [82] isolated 99 strains (PV1-99) from the mangrove forest of Kerala, India, and screened for the production of BSs through ODA. A yeast, namely *Geotrichum candidum* PV 37, was identified as the most promising candidate for BS production. FTIR analysis revealed the production of glycolipid BS by PV 37 with a yield of 2.8 g/L after completing an incubation of 120 hrs. BS also displayed stability under varied conditions (temperatures, pH, and salinity) and demonstrated effective antimicrobial (against MDR Gram-negative bacteria), anticancer, and antioxidant activities. This study is significant for current MDR pathogens and antimicrobial resistance. The remarkable activity of BS of PV 37 against MCF-7 (breast cancer cell line) also shows potential pharmaceutical applications. In another investigation, Nimsi et al. [83] reported a red pigmented yeast culture, *Rhodotorula paludegina* VA 242, from the Kerala mangroves for the production of BS. They selected seven morphologically distinct isolates through screening assays, namely ODT, parafilm M test, and EA. *R. paludegina* VA 242 showed high BS production potential and was of the glycolipid class. The BS also demonstrated remarkable antimicrobial and antioxidant activity and had cleaning activity (comparable to commercially available surfactant SDS), highlighting potential applications in pharmaceutical and detergent industrial sectors.

Loeto et al. [84] isolated the nine morphologically distinct halophilic yeasts from Makgadikgadi and Sua pans, as pristine and extreme environments in Botswana. All the strains were screened further for production of BS, among which two strains namely, *Rhodotorula mucilaginosa* SP6 and *Debaryomyces hansenii* MK9, exhibited high BS production abilities when the growth medium was supplemented with a seed powder of *Xanthocercis zambesiaca*. The seed powder was used as a novel alternative, inexpensive carbon substrate for the microbial fermentation process. Chemical characterization (through FTIR) of BSs suggested the production of RL from *R*. *mucilaginosa* SP6 and SL from *D. hansenii* MK9. Both BSs displayed some antimicrobial activity against selected pathogenic bacteria (*Proteus vulgaris, Escherichia coli, Klebsiella pneumoniae, Staphylococcus aureus, Micrococcus luteus*, and *Cryptococcus neoformans*) and fungi (*C. albicans* and *Aspergillus niger*). The liquid fraction residual of SL was mainly composed of carbohydrates, (32% w/w), lipids (1% w/w), and protein (15% w/w) and 497 ash (52% w/w) content. The SL-BS was the most effective against pathogenic strains, exhibiting high antimicrobial drug resistance. These findings open avenues for developing environmentally friendly antimicrobial drugs using inexpensive carbon sources to reduce monetary inputs in the BS production process.

Muthezhilan et al. [85] isolated 30 diverse yeast strains from saltern water and sediment samples from Kelambakkam Salterns-East Coast of Tamil Nadu, India. Morphologically, 30 marine yeast strains were isolated from saltern water and sediment samples using different media. Researchers used synthetic genetic array (SGA), yeast malt extract agar (YMA), yeast extract peptone dextrose (YPD), and Yeast Malt media (YM) to isolate those diverse yeast cultures through spread and pour plate techniques. The yeast strains were screened through ODT with five different oils (crude oil, olive oil, palm oil, coconut oil, and groundnut oil). Strains, namely AMBY101 to AMBY130, AMBY109, AMBY117, and AMBY124 were noticeable cultures for BS production. The strain (AMBY109) demonstrated significant production of BS using waste motor lubricant oil, crude oil, diesel, and kerosene, where significant EA was observed. Antimicrobial assays conducted for the purified BS from AMBY109 showed excellent antimicrobial potential. Balan et al. [86] collected sea sediment samples from 3 coastal regions (Porto Novo, Cuddalore, and Nagapattinam) of Tamil Nadu, India, and isolated 136 yeast strains and carried out routine screening assays to detect BS producers through tension-active properties, EA, and HA. Among all the isolates, *Cyberlindnera saturnus* SBPN-27 has the potential for a novel type of glycolipid BS production, which was termed Cybersan (Gal-Gal-Gal Heptadecanoic acid) with a yield of 2.13 g/L, reduced ST down to 28 mN/m with a CMC of 30 mg/L. Additionally, BS is stable at a broader range of pH and temperature. Cybersan showed multifunctional properties, including antibacterial activity against clinical pathogens with no cytotoxicity (3T3 fibroblast cells), signifying its suitability for biomedical applications.

Patel and Patel [87] isolated the BS-producing yeasts from five mangrove sites (Hazira, Mandroi, Mirzapur, Kantiyajal, and Machhad of Surat, Gujarat) and were screened further to produce BS using cottonseed oil as a substrate. A total of 24 yeast cultures were screened using the parafilm M test, ODA, and EA. Six yeast strains (Ky-46, Ky-53, Ky-54, Ky84, Ky-86, and Ky-87), showed positive results in all BS screening tests. Among all yeast strains, Ky-46 showed the highest activities, followed by Ky-86 for BS production. All the strains produced glycolipid type BS. Large-scale production of BSs from the yeast isolates can be made feasible using agro-industrial waste as a substrate.

The marine environment of the Antarctic region is a unique ecosystem of extreme cold, strong currents, with a high degree of isolation, and supports a distinct, rich biodiversity. Chaves et al. [88] explored the Antarctic region for BS producers. Yeast isolate, *Naganishia adellienses* L95, grew on lignocellulosic biomass as raw substrates to produce BS. Initially, xylose was supplemented as the carbon source with a crude extract of 4.27 g/L, and the BS produced showed substantial stability at 0 and 4 °C and 10% salt along with an alkaline condition. Correa et al. [89] also reported BS-producing yeast cultures from Antarctic environments. The yeast cultures, namely *Meyerozyma guilliermondii* L21, *C. glaebosa* L75, *Cryptococcus victoriae* L92, and *Leucosporidium scotti* L120, indicated substantial BS production abilities. Among the four strains, L75 and L120 showed the highest production of BS, 0.17 and 0.28 g/L, respectively. *Pseudozyma hubeiensis* SY62, a strain belonging to the basidiomycetous yeast, was isolated from a depth of 1156 m (Sagami Bay, Japan) in the habitat of a species of mollusk, *Calyptogena soyoae* (deep–sea, cold–seep clam) [90]. Usually, a combination of selective enrichment media encourages the isolation of marine microorganisms that have the capacity to produce commercially valued bioactive compounds. Researchers used olive oil and glucose to grow *P. hubeiensis* SY62 and produced glycolipid BS from the isolated yeast culture.

Leyton et al. [91] isolated a SL producing *Rhodotorula rubra* strain from the extract of *Macrocystis pyrifera*. SL was composed of carbohydrates (32% w/w), lipids (1% w/w), protein (15% w/w), and ash (52% w/w) and showed antibacterial activity against *E. coli* on *S. aureus*. Such BS can be employed as an antimicrobial agent to control pathogens associated with the food industry. Furthermore, a fascinating report was published for fragrant BS. Luepongpattana et al. [92] documented fatty acid-based amphiphilic molecules from *Aureobasidium pullulans* YTP6–14 (isolated from the Gulf of Thailand). ‘Massoia lactones' (lactonisedhydroxy fatty acids) is a liquid and available in nature with a creamy appearance and pleasant flavors (coconut and spicy). Massoia lactone exhibited a significant ST reduction (43.3 mN/m at 1 mg/mL). This is possibly the prime report recommending the production of fragrant BSs instead of an industrial level massoia lactone production from aromatic bark of *Cryptocarya massoy* (an endangered plant) that generally grows in rainforests. The diversity of BSs produced by *A. pullulans* was expanded, highlighting its potential contribution to green sustainable chemistry and rainforest conservation. Yeast cultures generally produce the SL class of BSs (acidic and lactonic), which is based on length, the composition of fatty acid chains, degree of saturation, and acetylation. Simple carbon sources (glycerol and glucose) and waste from food, oil, and agriculture wastewater were supportive in producing SLs from yeasts. The use of renewable wastes is significant in contributing to a circular bio-economy [93],[94]. In 2025, Matos et al. [95] isolated 146 yeast cultures from the microalga *Microchloropsis gaditana* (Olhão, Portugal), among which 60 isolates were tested for BS production through ODA and EI. All yeast strains showed positive results with considerable BS activity in ODA with minor differences.

Cheap or renewable substrates have been utilized in the production of BSs/BEs from marine fungi. Several inexpensive agricultural industry-based substrates have been employed to produce BS from marine microorganisms. Marques et al. [9] formulated CSL and oil–based medium for BE production from *Mucor circinelloides* UCP0001, which was isolated from mangrove sediments. Moreover, the use of renewable and affordable substrates enables affordable biosynthesis of surface–active agents. The production of BSs/BEs from microbial sources is dependent on several parameters of substrates such as their stability, availability, abundance, form, and concentrations [96]. Hydrocarbons are usually reported as the sole source for the production of BS from marine microbes. Agricultural waste like date molasses, cassava waste, orange peel, CSL, and sugarcane bagasse is helpful in cost reductions, large–scale substrate availability, maintaining intact functional properties of BS without harming the environment [97]–[99]. Emulsifying properties, stability studies (pH, temperature, and salinity), safety, and properties of BE were evaluated successfully. The authors recommended CSL (4%) and waste soybean oil (3%) to produce 2.69 g/L of BE. Glycolipoprotein-based BE comprising carbohydrates (28%), protein (14%), and lipids (40%) is not toxic to *Artemia salina* (brine shrimp) and *Chlorella vulgaris* (green microalga). Mannosylerythritol lipids (MEL) have been produced by the marine yeast *Moesziomyces aphidis* XM01, which was isolated from mangroves (Hainan Province of China). The strain was grown in soybean oil, and the BS produced was utilized further for the preparation of nano-micelles, which can be employed for pharmaceutical and cosmetic-related applications [100].

Like bacteria and yeast cultures, fungal strains can also use crude oil to produce BS. Pitocchi et al. [101] isolated two fungal strains, *Aspergillus terreus* MUT 271 and *Trichoderma harzianum* MUT 290, from a Mediterranean marine site chronically pervaded by oil spills, using crude oil as the sole carbon source. These strains were investigated as producers of BSs, which was appropriate to solubilize organic molecules as a preliminary step to metabolize them. Both fungi secreted low molecular weight proteins identified as cerato-platanins, which are small, conserved, hydrophobic proteins that are included among the fungal surface-active proteins. Both proteins were able to stabilize emulsions, and their capacity was comparable to that of other BS proteins and to commercially available surfactants. The cerato-platanin from *T. harzianum* (*Th*CP) reduced ST to a larger extent than the similar protein from *A. terreus* (*At*CP) and other amphiphilic proteins from fungi. The culture broth of *A. terreus* (2.7 ± 0.3 mg/L) and *T. harzianum* (1.7 ± 0.5 mg/L) yielded a protein with considerable emulsifying activities. Both cerato-platanins (hydrophobins) were able to transform a hydrophilic surface into a hydrophobic surface efficiently and to form a stable layer that was not removable even after surface washing [101]. Marine sponges are invertebrates that come under the phylum Porifera. These sessile and filter-feeding living entities are found at various depths (intertidal zones to abyssal depths). Sponges contribute to marine ecosystems, especially to bio-erosion (eroding hard substrates and coral reefs to produce sediment), stabilization of several substrates, and the creation of reefs. Additionally, sponges serve as a food source and a healthy environment for several marine organisms. Microorganisms associated with sponges have been reported to produce several bioactive compounds, including BSs/BEs. Fungi obtained from extreme marine systems possess significant biotechnological potential. Nogueira et al. [102] isolated 75 filamentous fungi from the marine samples (Paraná, Brazil) and explored them for the production of bioemulsifying agents along with the bioremediation potential of petroleum-derived compounds. Three strains, including *Penicillium* spp. FM16, FM02, and *Trichoderma* sp. FM14, were found to be useful for bioremediation processes, particularly at marine sites polluted with petroleum-based compounds. da Silva et al. [103] isolated fungal cultures from deep marine sediments collected from three islands. About 68 fungi belonging to the Ascomycota phylum were isolated, and the cultures were assessed for BS production through EA, where *A. psychrotrophicus* UFMG 19617 was an effective BS producer.

Kiran et al. [104] isolated the endosymbiotic fungus *Aspergillus ustus* (MSF3) from the marine sponge *Fasciospongia cavernosa*, collected from the peninsular coast of India. This strain produced a high yield (based on the EA) of BS in Sabouraud dextrose-based fermentation broth. Optimized bioprocess conditions assisted the production of BS (high EA) at pH 7.0, temperature 20 °C, and salt concentration of 3% with a carbon–nitrogen ratio of 3:2, with 75% E_24_. Additionally, glucose and yeast extracts proved to be supportive carbon and nitrogen sources, respectively. The BS produced by MSF3 was partially characterized as a glycolipoprotein based on macromolecular estimation and thin-layer chromatography (TLC) analysis. The partially purified BS exhibited broad–spectrum antimicrobial activity. Additionally, the strain MSF3 has potential applications in MEOR [104]. The same research group [105] explored BS production of *Aspergillus* sp. MSF1, which was associated with the marine sponge (*Dendrilla nigra*). Several screening protocols (HA, ODT, DCT, and EA) carried out by the researchers indicated BS production abilities by *Aspergillus* sp. MSF1. The submerged BS fermentation process under optimized conditions improved BS production, which was identified as rhamnolipid/s (RLs), confirmed through several analytical techniques (TLC, FT-IR, and HPLC). EA of CFS and extracts obtained using different solvents were exceptional compared to the chemical surfactants (SDS and Tween 80). The RL was found to possess antimicrobial activity against selected yeast pathogens (*C. albicans*) and Gram-negative bacteria.

Teixeira et al. [106] isolated 116 fungal cultures from the sediment samples (Boeckella Lake, Antarctic Peninsula) and assessed them for BS production. Fungi belonging to Ascomycota, Basidiomycota, and Mortierellomycota were observed. Among all isolates, 14 fungi showed considerable EA, which could be explored in further varied biotechnological applications. Fungal genera Cosmospora, Pseudogymnoascus, Ramgea, and a few yeast strains (*Cystobasidium* and *Thelebolales* sp.) were reported for BS production for the first time. Bioprospecting of marine metagenomics is remarkable in discovering innovative BSs/BEs molecules to overcome the challenges associated with low yield and explore them for a range of applications in diverse industrial sectors [107],[108]. Numerous studies document production of around 10 to 200 g/L yield of BS during the yeast fermentation process [76], where *Candida*, *Yarrowia*, *Aspergillus*, and *Cunninghamella* species have been reported frequently to produce BS from other environments. Generally, RL, SL, MEL, and glycoprotein-based BS have been reported from non-marine microbial sources. Fungi and yeasts found in marine habitats usually produce lower quantities ≈ 10 g/L of BSs/BEs; however, they frequently show salt tolerance and activity in extreme environmental conditions. The marine *Candida, Yarrowia*, *Rhodotorula, Cystobasidium*, *Thelebolales, Aspergillus, Mucor*, and *Trichoderma* have been reported frequently for glycolipid types like RL, SL, and glycoprotein type BS production. Moreover, novel type glycolipids, namely Cybersan (trigalactomargarate) and Massoia lactones, have been reported from marine yeast strains. The yields of BS depend on the strain, type of substrate, fermentation conditions, and optimization parameters. A summary of BSs/BEs produced by marine yeast and fungi with their potential applications is shown in Table 1.

**Table 1.** Biosurfactants (BSs)/Bioemulsifier (BEs) produced by marine yeast and fungi and their potential applications.

Microorganism	Class	Sampling site/source	Methods for extraction	Properties of surface-active agent				Potential application (of the biosurfactants unless specified otherwise)	Reference
				Surface tension (mN/m)	Critical micelle con. (CMC)	Emulsification (%)	Yield		
*Rhodotorula mucilaginosa*, *R. diobovata* and *M. guillier-mondii*	Sophorolipids, glycolipids	Associated with microalga *Microchloropsis gaditana*, *Portugal*	No extraction; only cell-free supernatant evaluated using oil displacement and emulsification index	--	--	--	--	Biofuels, oleochemicals and cosmetics; antifungal activity	[95]
*Yarrowia lipolytica* LMS 24B	Glycoprotein	Associated with zoanthids, Brazil	--	--	15 mg/mL	53.14 ± 4.3	--	--	[78]
*Trichoderma* sp. FM14, *Penicillium* sp. FM02 and FM16	Keratoplatanins	Marine samples collected on the coast of the states of Paraná, Brazil	No extraction; cell-free supernatant evaluated by emulsification index	--	--	17.5	--	Bioremediation process	[102]
*Thelebolus balaustiformis* UFMGCB 19606, *Antarctomyces psychrotrophicus* UFMGCB 19608, *Antarctomyces psychrotrophicus* UFMGCB 19617, *Penicillium. cf. palitans* 19671		Three islands in the South Shetland Islands archipelago, maritime Antarctica	Cell-free supernatant used for biosurfactant activity assessment	--	--	53.04, 63.35, 81.38, and 63.71 respectively	--	*A. psychrotrophicus* UFMG 19617 demonstrated similar biosurfactant activity as compared to the commercial surfactant SDS	[103]
*Antarctomyces psychrotrophicus* (eight isolates)*, Cosmospora* sp. (two isolates)*, Pseudogymnoascus* sp. (two isolates)*, R. cf. ozimecii* (one isolate) and *Thelebolus* sp.	--	30 cm long sediment cores from the west and south-east shores of Boeckella Lake, Hope Bay, North-east Antarctic Peninsula	Production was evaluated by EI_24_% by the cell-free supernatant only	--	--	78.60 ± 7.04 (three isolates of *Antarctomyces psychrotrophicus*)	--	As a biodegradable and sustainable alternative to synthetic surfactants	[106]
*Rhodotorula paludigena* VA 242	Glycolipid	Mangroves of Central Kerala, India	Acid precipitation, solvent extraction n-hexane:methanol:wa-ter (1:6:3)	--	--	49.7		Antioxidant, antibacterial activity, Detergent additive	[83]
*Moesziomyces aphidis* XM01	Mannosylerythritol lipids (MEL)	Mangrove systems in Hainan Province of China	Acid precipitation, solvent extraction using chloroform: methanol (2:1)	--	0.79 mg/L (≈1.12 × 10 –3 mmol/L)	--	113.6 ± 3.1 g/L within 8 days	MEL Nanomicelles as pharmaceutical carriers for hydrophobic drugs; antibacterial activity	[100]
*Candida bombicola*	Glycolipid	Not mentioned. Preparation of BS samples in marine synthetic samples	Acid precipitation, solvent extraction (methanol)	29 (flask), 33 (1.2 L bioreactor), 31 (3 L bioreactor), 30 (50 L bioreactor)	0.5%	47 (flask), 30 (1.2 L bioreactor), 11 (3 L bioreactor), 58 (50 L bioreactor)	221.9 g/L	Food emulsions	[81]
*Rhodotorula mucilaginosa SP6* and *Debaryomyces hansenii MK9*	Rhamnolipid Sophorolipid	Makgadikgadi and Sua pans, Botswana	Acid precipitation, Solvent extraction (ethyl acetate)	26.1 31.4	--	91.1 56.1	--	Antimicrobial activity	[84]
*Rhodotorula rubra*	Sophorolipid	Seaweed tissue, Puerto Montt, Chile	Cell-free supernatant evaluated only	--	--	--	0.1 g/L	Antibacterial activity	[91]
*Leuconeurospora sp*. L101, *Candida guilliermondii* FTI 20037, *Naganishia adeliensis* L95, *Naganishia adeliensis* L110, *Papiliotrema laurentii* L62, *Rhodotorula mucilaginosa* L65, *Tremella indecorata* L99	Glycolipid	Marine and terrestrial samples from Antarctic region, Brazil	Acid precipitation by HCl followed by solvent extraction by chloroform: methanol (2:1 and 4:1), hexane: ethyl acetate (7:3), ethyl acetate	--	--	36.89 ± 0.11 56.40 ± 0.03 55.69 ± 0.02 42.67 ± 0.12 58.48 ± 0.04 63.65 ± 0.03 44.97 ± 0.04	4.27 g/L (by the chloroform-methanol of 4:1)	Tolerance to variable pH and high salt concentrations make them suitable in bioremediation, also in pharmaceutical and cosmetic industries	[88]
Yeast strains	Glycolipid	Hazira, Mandroi, Mirzapur, Kantiyajal and Machhad: five mangrove sites of Surat, Gujarat, India	No extraction done; cell-free supernatant was evaluated	--	--	--	--	Yeast strains may be used to utilise agro-industrial wastes to produce biosurfactants	[87]
*Aspergillus terreus* MUT 271 *Trichoderma harzianum* MUT 290	Cerato-platanins (surface-active protein)	Marine site in the Mediterranean affected by oil spills, Italy	Ultrafiltration (30 kDa filter), concentration and dialysis in sodium phosphate buffer (10 mM, pH 7.0)	56.3 36.4	0.12 mg/mL (9 × 10^−6^ M), 0.10 mg/mL (8 × 10^−7^ M),	56, 44 (0.05 mg/ml protein concentration) 83, 70 (0.1 mg/ml)	2.7 ± 0.3 mg/L, 1.7 ± 0.5 mg/L	Showed good surface tension reduction and emulsification properties thus has use in medical, petrochemical, agriculture, food, textile, cosmetic, industies	[101]
*Mucor circinelloides* UCP0001	Glycolipoprotein	Mangrove sediment, Rio Formoso municipality, Pernambuco-PE, Brazil	Cell-free supernatant was used for EI; Bioemulsifier was precipitated by ethanol (1:2 v/v), kept undisturbed for 24hr at 4 °C, centrifuged, evaporated (excess ethanol removal), and lyophilised	--	--	100 (motor oil)	2.69 g/L	Bioemulsifier	[9]
*Aureobasidium pullulans* YTP6 –14	Massoia lactones	Seawater from the coast of Koh Sichang, Chonburi Province, Gulf of Thailand	Solvent extraction by ethyl acetate, evaporation under vaccum	31.6	39 mg/L	--	1.3 g/L (optimized condition: 2.5% glucose(w/v) + 2.5% glycerol(v/v)	Fragrant biosurfactant	[92]
Yeast strains	--	Kelambakkam salterns - East coast, Tamil Nadu, India	Acid precipitation and then solvent extraction with chloroform: methanol (2:1)	--	--	--	--	Antimicrobial activity	[85]
*Aspergillus* sp. MSF1	Rhamnolipid	Associated with marine sponge *Dendrilla nigra*, Tamil Nadu, India	Acid precipitation, followed by solvent extraction (ethyl acetate, diethylether, dichloromethane).	28	--	11.4 (cell-free supernatant), 32 (diethyl ether extract)	9.46 mg/L (diethyl ether extract)	Antimicrobial activity	[105]
*Yarrowia lipolytica* IMUFRJ 50682	--	--	--	19.5	--	81.3		--	[79]
*Pseudozyma hubeiensis* SY62	Mannosylerythritol lipids	Deep sea, Calyptogena soyoae collected from Sagami bay. Japan	Solvent extraction by ethyl acetate	--	--	--	129 ± 8.2 g/L	--	[90]
*Aspergillus ustus* MSF3	Glycolipoprotein	Endosymbiote of marine sponge *Fasciospongia cavernosa*, Tamil Nadu, India	Acid precipitation, liquid–liquid extraction method (both cell free supernatant and cell pellet), followed by solvent extraction (ethyl acetate, diethyl ether, dichloromethane)	--	--	42.8	--	Antimicrobial activity and applications for Microbial enhanced oil recovery	[104]

-- : Information not mentioned.

## 6. Conclusion and future prospects

Compared to their terrestrial counterparts, marine-origin BSs/BEs producing yeasts and fungi remain significantly underexplored. The novelty, with respect to structures and functional properties of BSs/Bes, originating from marine microbes, is quite interesting. Some non-marine yeast and fungi reported for BS production may be associated with some pathogenicity issues. The chance of contamination is reduced while working with marine microbes compared to non-marine cultures. Limited literature is available for marine yeasts and fungi compared to other sources, and their molecular genetics traits involved in the production of surface-active/emulsifying agents need to be better explored. The metagenomics approach supports the discovery of novel BSs/BEs to overcome leading challenges, such as low yields Some success stories have satisfactorily depicted achievements, though challenges are expected in the near future on production technology involved in BS production. The wide range of possible applications can be achieved by investigating the underlying molecular mechanisms involved in the production of BSs/BEs, which is the main requirement. Over–producing strains or modification of production procedures can be improved from an extensive knowledge of the genetic makeup and regulatory mechanisms involved in the production of BSs/BEs. This may lead to achieving higher yields with lower production costs and environmentally friendly manufacturing practices.

## Use of AI tools declaration

The authors declare they have not used Artificial Intelligence (AI) tools in the creation of this article.
